# Posturography techniques to identify balance problems in elderly individuals with vestibulopathy – a comparison study

**DOI:** 10.1016/j.bjorl.2025.101743

**Published:** 2025-11-26

**Authors:** Cibele Brugnera, Raquel Mezzalira, Lucas Resende Lucinda Mangia, Luiz Cezar da Silveira, Bruno Souza, Roseli Saraiva Moreira Bittar

**Affiliations:** a Faculdade de Medicina da Universidade de São Paulo, Departamento de Otorrinolaringologia, São Paulo, SP, Brazil; b Universidade Estadual de Campinas (UNICAMP), Departamento de Otorrinolaringologia, Campinas, SP, Brazil; c Universidade Federal do Paraná (UFPR), Departamento de Otorrinolaringologia, Curitiba, PR, Brazil

**Keywords:** Postural imbalance, Posturography, Falls, Elderly

## Abstract

**Objective:**

To compare the postugraphic outcomes of two different methods in four basic postural conditions.

**Methods:**

The individuals with vestibular disorders were divided into two subgroups according to their age: more than 60-years old and aged between 18 and 60. All the participants underwent to computerized dynamic posturography (EquiTest System®) and a mobile posturography (Vertiguard System®) in four test conditions: (1) Eyes open and stable surface, (2) Eyes closed and stable surface, (4) Eyes open and unstable surface, (5) Eyes closed and unstable surface.

**Results:**

The elderly group presented worse overall outcomes in computerized dynamic posturography compared to controls. These outcomes were not different between groups when mobile posturography was used. For both methods, the results were not different between groups when the Conditions 1, 2 and 5 were evaluated separately. A significantly higher proportion of elderly subjects exhibited alterations in C4 during mobile posturography.

**Conclusion:**

Computerized dynamic posturography was overall more sensitive at detecting abnormal postural sway in elderly individuals with vestibular disorders. In mobile posturography, Condition 4 also seemed particularly sensitive at detecting postural instability.

**Level of evidence:**

2.

## Highlights

• Elderly people have less ability to maintain posture.
• Postural instability can lead to imbalance and falls.
• Computerized dynamic posturography was more sensitive to detect imbalance in elderly.
• Mobile posturography can identify postural instability in the elderly in Condition 4.

## Introduction

Balance control evaluation of the elderly is useful to document overall postural stability and predict disturbances that might predispose to falls.^1^, ^2^, ^3^ Posturography is a noninvasive quantitative technique that measures shift in the center of mass under static or dynamic conditions. A range of equipment is currently available, with technical and practical particularities. The force platforms are highly sensitive devices, but more costly. An alternative is a system consisting of a belt with sensors adjusted to the hip ‒ a mobile posturography.

Using less expensive, simpler, and easy-to-transport equipment could help implement a broader population evaluation of balance, which might benefit particularly aging individuals. Easier access to balance assessment in primary care could assist healthcare professionals in preventing falls and their common and potentially serious consequences. The quest for a reliable method to assess and triage balance in different populations is an underexplored yet important field of research. To achieve this goal, comparing the performance of different posturographic devices in the four basic static postural positions is likely a good starting point.

This study compares the posturographic outcomes of a force platform and a belt adjusted to the hip in the four basic postural conditions. Secondly, we explore the results obtained according to age group.

## Methods

This cross-sectional study was approved by the Institutional Ethics Committee (nº 4.834.397). Inclusion criteria were age > 18, previous diagnosis of vestibular dysfunction, and current complaints of static or dynamic imbalance. Those with neurological, cognitive, or musculoskeletal conditions affecting the ability to perform the posturography were excluded.

The overall sample comprised 108 individuals. They were divided into two subgroups according to their age: 69 elderly subjects, who were more than 60-years-old, and controls, aged between 18 and 60. The main underlying mechanism of their vestibular dysfunction was determined and classified into five categories: vascular (34%), metabolic (23.4%), traumatic (25.5%), neurotological (12.7%), and ototoxicity (4.2%).

### Intervention

All the participants underwent two different posturography assessments on the same day: Computerized Dynamic Posturography (CDP), using the EquiTest System® (NeuroCom International, Clackamas, United States) and a Mobile Posturography (MP), using the Vertiguard System® (Zeisberg GmbH, Metzingen, Germany).

The protocol used during the CDP was the sensory organization test, which quantifies the anteroposterior projection of the center of mass in the platform. With the subject standing on the platform, four test conditions were recorded for 20 seconds each: Condition 1 (C1) – Eyes open, fixed platform; Condition 2 (C2) – Eyes closed, fixed platform; Condition 4 (C4) – Eyes open, sway-referenced platform; Condition 5 (C5) – Eyes closed, sway-referenced platform.

MP consisted of a belt adjusted to the patient’s hip, with sensors capable of detecting anteroposterior and lateral displacements. The participants were asked to keep their feet aligned with their shoulders and gaze at a target placed three meters ahead for 60 seconds. A firm foam (25 k/m^3^ density) was placed beneath their feet to make the surface unstable when needed. Four similar conditions were tested: Condition 1 (C1): Both feet on stable ground, eyes open; Condition 2 (C2): Both feet on stable ground, eyes closed; Condition 4 (C4): Both feet on foam rubber (unstable), eyes open; Condition 5 (C5): Both feet on foam rubber (unstable), eyes closed.

The results of each condition and method were compared with reference data provided by the manufacturers and classified as “normal” or “abnormal”.

### Statistical analysis

The overall sensitivity of each test and for each age group was obtained. To test for normality, the Kolmogorov-Smirnov test was used. The Chi-Square test with Yates correction was performed to compare qualitative data. Numerical variables were compared using the *t*-test. The significance level was set at 0.05.

## Results

**Fig. 1:**
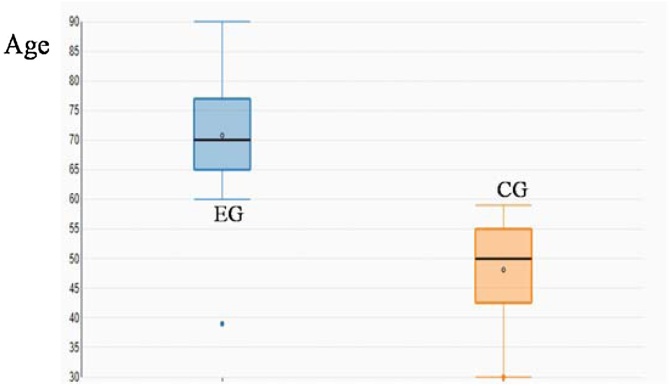
Age distribution in the Elderly Group (EG) and Control Group (CG).

The elderly group comprised 69 patients (33 male and 36 women; mean age 70.78 ± 8.42 years-old), whereas the control group included 39 patients (18 men and 21 women; mean age 48.1 ± 9.02 years-old). Their age was significantly different (p < 0.0001, *t*-test, Fig. 1).

The distribution of the etiological mechanisms of the vestibular symptoms was not different between elderly and control groups (Table 1).

**Table 1:** Distribution of the etiologies found in percentages in the elderly and control groups and their significance (p).

	Elderly (%)	Control (%)	p-value
Metabolical	0.25	0.21	1
Neurological	17.80	0.52	0.38
Ototoxic	0	0.1	0.15
Traumatic	17.80	36.84	0.18
Vascular	39.20	26.31	0.53

The CDP and the MP showed an overall sensitivity of 97% and 75% in the elderly group, respectively. This result reached statistical significance (χ^2^ = 13.7329, p = 0.0002; Yates = 11.9628, p = 0.00054). The sensitivity was not significantly different between these methods when assessing the control group (χ^2^ = 2.2792, p = 0.1311; Yates = 1.5828, p = 0.2083).

The elderly group presented worse overall outcomes in CDP compared to controls (χ^2^ = 9.2011, p = 0.0024; Yates = 7.2241, p = 0.007193). However, these outcomes were not different between groups when MP was used (χ^2^ = 1.5436, p = 0.214).

For both methods, the results were not different between groups when the conditions C1 (PDC: χ^2^ = 0.0098, p = 0.9212; and MP: χ^2^ = 1.9598, p = 0.1615) and C2 were evaluated separately (PDC: χ^2^ = 2.7229, p = 0.9891; and MP: χ^2^ = 0.1609, p = 0.6883). Nonetheless, the proportion of individuals in the elderly group with abnormal results in C4 during MP was significantly higher (χ^2^ = 7.5251, p = 0.00608; Yates = 6.3595 p = 0.01167). In C5, the proportion of subjects with alterations was significantly higher in controls when compared to the elderly group (χ^2^ = 4.022, p = 0.04429**)**; after Yates (2.8033, p = 0.09407) the results were not different.

There was no difference in the overall results obtained in CDP according to sex when the study groups were evaluated separately (elderly group: χ^2^ = 0.2146, p = 0.6432; control group: χ^2^ = 0.4145, p = 0.5196). For the elderly group, women and men also did not present significantly different outcomes in MP (χ^2^ = 0.5132, p = 0.4737). Among controls, women tended to show worse results in MP (χ^2^ = 0,4148, p = 0,0416), although this difference was not confirmed after the Yates correction.

## Discussion

Falls are a mounting issue in developing and developed countries, and are associated with enormous healthcare costs and great morbidity, particularly in the elderly. The diagnosis of balance problems and identification of potential risk factors in populations at higher risk is the better option to tackle the problem.^4^ The easier and sooner the affected individuals are diagnosed and treated, the better the health-related outcomes at both the individual and community levels.^5^, ^6^, ^7^

This study aimed to evaluate the performance of different devices used to assess balance control. In particular, the MP could be a simple, portable, and practical tool to study postural control among the elderly. This device differs from the well-established CDP because it measures not only anteroposterior sways but also lateral displacements of the center of mass. Another particularity of the MP is the unique capacity to assess the balance control during body movements.^8^

CDP presented better overall performance in both study groups, and the elderly showed worse results than the control group. The sensitivity of MP was similar between the elderly and controls. These findings suggest that the CDP is better at identifying balance problems in individuals with vestibulopathy, regardless of their age.

Nonetheless, in Condition 4, with the patient on unstable ground with eyes open, the MP showed significantly poorer results among the elderly. The CDP was not able to show any differences between the groups in particular conditions. As previously reported, an increase in lateral sway in senescence and among subjects with higher risks of falls could explain these findings. The MP detected these increased lateral displacements that likely go unnoticed with CDP.^9^

Medical literature often ascribes greater body oscillations among the elderly to structural damage of the vestibular maculae.^10^^,^^11^ However, this is not necessarily due to damage isolated to the saccule and utricule, as multiple sensory networks converge and are integrated into the central nervous system to enable balance control. Hence, in Condition 4, vestibular information works with vision and proprioception to maintain postural stability. The elderly often have comorbidities causing dysfunctions in one or more of these sensory pathways that could cause greater posturographic impact when a vestibular problem is present.^12^ Loss of muscle mass and motor coordination with aging could also impair balance in unstable conditions in older individuals with vestibulopathy and help explain the findings.^9^

In Condition 5, regarded as typically vestibular, the control group tended to present poorer postural control than the elderly group, even though this statistical difference did not persist after the Yates correction. The presence of midlife women among controls might have biased the results towards a worse performance in this condition. In an epidemiological study in the same city where this work was done, the prevalence of dizziness demonstrated two peaks. The first of up to 49%, in individuals between 45–55 years, notably women, and the second, of 44%, among the elderly. Thus, dizziness affected preferably middle-aged women, who are particularly prone to the effects of hormonal fluctuation and transition on the vestibular system.^13^ Not surprisingly, the mean age of the control group in this study coincides with the menopausal transition.

Although not significant after statistical correction, control women tended to show worse posturographic results than the elderly women. This age disparity was not observed among men. Altogether, these findings could point to a specific impact of the hormonal changes on the balance control of adults which is not seen in older individuals.^14^, ^15^, ^16^, ^17^, ^18^

In brief, one could assume that the control group could include more patients with “pure” vestibular abnormalities (with clearer alterations in C5). On the other hand, the elderly group could encompass patients with multifactorial balance problems, with marked postural sway in C4.

As a result we used only the similar and comparable conditions of the CDP and the MP. This shortened evaluation seemed to be useful for detecting balance problems in patients with vestibulopathy. Future population studies could investigate their discriminative performance as triage methods, particularly in patients with an increased risk of falls. Yet, we must highlight that a complete sensory integration test in CDP and the MP (Vertiguard®) uses six and 13 conditions to investigate the balance control, respectively. The results also match current evidence showing a marked prevalence of imbalance due to vestibular alterations among midlife women, whereas in older individuals this manifestation is usually multifactorial. Hence, evaluation of Condition 4 using MP might be promising in detecting disturbances in postural stability in the elderly.

## Conclusion

The CDP was overall more sensitive than the PM at detecting abnormal postural sway in elderly individuals with vestibulopathy. In PM, Condition 4 also seemed particularly sensitive at detecting postural instability in these individuals.

## ORCID ID

Cibele Brugnera: 0009-0009-3096-4250

Raquel Mezzalira: 0000-0002-0279-8007

Lucas Resende Lucinda Mangia: 0000-0003-3443-3640

Luiz Cezar da Silveira: 0009-0002-1688-0199

Bruno Souza: 0000-0002-9693-7329

Roseli Saraiva Moreira Bittar: 0000-0001-8731-8908

## Funding

There is no financial or material support.

## Declaration of competing interest

The authors declare no conflicts of interest.
